# Global Research Trends on Water Contamination by Microorganisms: A Bibliometric Analysis

**DOI:** 10.3390/ijerph22071128

**Published:** 2025-07-17

**Authors:** Zoila Isabel Cárdenas Tirado, Isaías Wilmer Duenas Sayaverde, Rosario del Socorro Avellaneda Yajahuanca, Sdenka Caballero Aparicio, Kelly Myriam Jiménez de Aliaga, Edo Gallegos Aparicio, Maria Antonieta Rubio Tyrrel, Maria do Livramento Fortes Figueiredo, José Wicto Pereira Borges, Rosilane de Lima Brito Magalhães, Denise Andrade, Daniela Reis Joaquim de Freitas, Ana Raquel Batista de Carvalho, Maria Eliete Batista Moura

**Affiliations:** 1 Nursing Department, Universidad Nacional Autonoma de Chota (UNACH), Chota 06121, Peru; zcardenas@unach.edu.pe (Z.I.C.T.); iduenias@unach.edu.pe (I.W.D.S.); rsavellaneday@unach.edu.pe (R.d.S.A.Y.); kmjimeneza@gmail.com (K.M.J.d.A.); 2 Faculty of Health Sciences, Universidad Andina del Cusco, Cusco 08002, Peru; sdenka_ca@hotmail.com (S.C.A.); egallegos@uandina.edu.pe (E.G.A.); 3 Nursing Department, Federal University of Piaui, Teresina 64049-550, Brazil; tyrrell2004@hotmail.com (M.A.R.T.); liff@ufpi.edu.br (M.d.L.F.F.); wictoborges@ufpi.edu.br (J.W.P.B.); rosilane@ufpi.edu.br (R.d.L.B.M.); danielarjfreitas@ufpi.edu.br (D.R.J.d.F.); 4 Ribeirão Preto College of Nursing, University of São Paulo, São Paulo 14040-902, Brazil; dadrade@eerp.usp.br; 5 Medicine Department, Uninovafapi University Center, Teresina 64073-505, Brazil; ana.raquel@uninovafapi.edu.br

**Keywords:** microorganism, waterborne pathogens, drinking water, water quality, water pollution, water treatment, disinfection, filtration, public health, waterborne diseases

## Abstract

Water is an essential resource for life; however, the quality of available water on the planet has been compromised due to various factors, including microbiological contamination. Objective: To analyze the global scientific production of microbiological water contamination using bibliometric methods. Method: A search for scientific articles was conducted using the advanced query function in the Web of Science™ database, specifically in its core collection, on 26 February 2025. Data from 2000 articles were analyzed using the Bibliometrix package in R (version 4.2.1) and the Biblioshiny application (version 2.0). Results: The evaluated articles were published between 1952 and 2025, with a peak in publications in 2022. The journal *Water Research* stood out as the most relevant, publishing 128 articles. The Egyptian Knowledge Bank was identified as the most productive institution, while China had the highest number of contributing authors. The most cited article received 475 citations. Additionally, KeyWords Plus™ highlighted the focus of the studies on ecological and biotechnological methods for contaminant removal, as well as the presence of waterborne pathogens and their inactivation methods. Conclusions: The results show a growing interest in the development of ecological and biotechnological methods for contaminant removal and pathogen inactivation in water. The integration of artificial intelligence with real-time monitoring systems emerges as a promising strategy for improving water quality management. These findings highlight the relevance of the topic for public health and health education.

## 1. Introduction

Water is an essential resource for life, playing a fundamental role in maintaining human health, ecosystem balance, and socioeconomic development [1]. However, water quality has increasingly been compromised due to various factors, including microbiological contamination, which poses a significant threat to public health [2]. Microorganisms such as bacteria, viruses, protozoa, and fungi are frequently found in water bodies and can cause severe infectious diseases, such as cholera, viral hepatitis, and cryptosporidiosis, especially in regions with inadequate or non-existent sanitation [3].

Global concern about water quality and the impact of related diseases is reflected in the United Nations (UN) Sustainable Development Goals (SDGs). Specifically, SDG 6 aims to “ensure availability and sustainable management of water and sanitation for all,” proposing among its targets the reduction in water pollution and improvement of water quality by 2030. This commitment reinforces the need for robust strategies for monitoring and controlling waterborne pathogens, particularly in vulnerable regions where the lack of sanitation infrastructure exacerbates the risk of waterborne diseases [4,5].

Moreover, climate change and accelerated urbanization have intensified pressure on water resources, aggravating risks associated with microbiological contamination. The impact of climate variations on the presence of aquatic pathogens represents an additional challenge for water quality management, requiring adaptation strategies that consider both ecological and public health aspects [1,6].

Technological advances in recent decades have provided effective tools for microbiological monitoring, such as quantitative PCR (qPCR), which enables the identification of pathogens with high precision, even at low concentrations. Modern techniques, such as next-generation sequencing, have revolutionized the study of aquatic microorganisms, offering detailed information about the composition and dynamics of microbial communities [7]. However, challenges remain, such as the increase in antimicrobial resistance and the presence of emerging pollutants, which demand integrated and transdisciplinary approaches to contamination control [3].

In this context, bibliometric analysis emerges as an essential tool for mapping global research trends on water contamination, identifying the main research topics, international collaboration networks, and knowledge gaps [8,9]. Recent studies have used this approach to understand the evolution of research in related areas, such as wastewater reuse and effluent treatment, providing support for the formulation of public policies aimed at infection control and the sustainability of water resources [10].

Thus, the present study aims to analyze global scientific production on microbiological water contamination using bibliometric methods.

## 2. Materials and Methods

This study is characterized as bibliometric research with a quantitative and descriptive approach, based on documentary analysis. Bibliometrics, as established by Pritchard (1969), refers to the application of statistical and mathematical methods to evaluate scientific production and academic communication, providing support for the dissemination of knowledge and the assessment of the productivity of authors, institutions, and countries [11].

To ensure the transparency and reproducibility of the results, this study followed the guidelines of the Preferred Reporting Items for Bibliometric Analysis (PRIBA), adapted from the PRISMA checklist, as proposed by Koo and Lin (2023) [12]. The bibliometric data collection was carried out on 26 February 2025, using the Web of Science (WoS) platform, one of the largest and most reliable academic databases, encompassing approximately 1.9 billion cited references in over 171 million records. This choice was justified by the broad coverage and high quality of the indexed journals, which are essential to ensuring the robustness of the results [13].

To formulate the search strategy, descriptors from the Medical Subject Headings (MeSH) were used, along with Boolean operators and wildcard characters, aiming to optimize retrieval and specificity of the results. To specify the topic, increase precision, and reduce false positive results, the present study considered only article titles. The literature has already reported that specific title searches increase retrieval and specificity while generating minimal sensitivity loss compared to searches that include all fields [14,15].

Only original articles published up to 26 February 2025 were included. Articles outside the scope of the research, review articles, opinion articles, reflection articles, editorials, and case studies were excluded.

**Figure 1:**
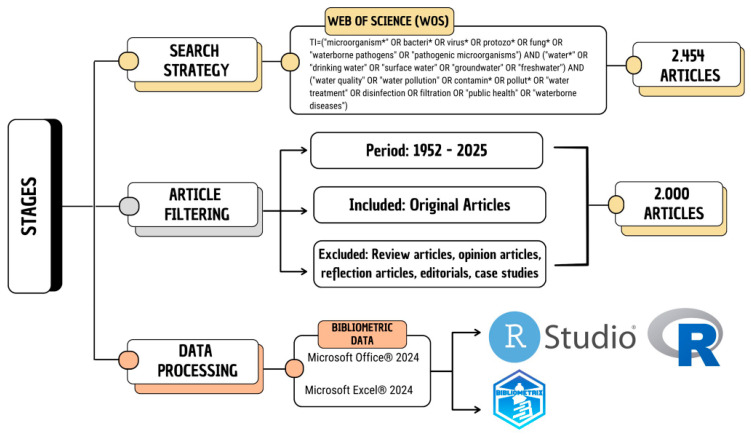
Search strategy used and selection of articles included.

The initial search in the database resulted in 2475 articles. After filtering was conducted and the criteria were applied, 2000 articles remained, as described in Figure 1. All available information from these articles was downloaded in a text file format for analysis. Bibliometric research also describes these data as “metadata” [11,16].

The metadata were imported into the R Studio Desktop Software, version 2023.03.0+368 (©Posit Software, Boston, MA, USA, 2023), linked to the R Software, version 4.2.3 (The R Foundation, Vienna, Austria, 2023). For the analysis, we used the Bibliometrix R package (version 4.2.1) (© K-Synth Srl, Academic Spin-Off of the University of Naples Federico II, Naples, Italy, 2023) and the Biblioshiny application (version 2.0), which provides a web interface for Bibliometrix [16].

Bibliometrix is an open-source tool designed to perform comprehensive scientific mapping analysis of scientific literature, programmed in R to be flexible and facilitate integration with other statistical and graphical packages [16].

The analysis allowed the visualization of article production according to the publication year, the scientific journals that published the most and those most cited according to Bradford’s Law [17], author productivity over time, the most productive institutions and countries, the most cited articles, the conceptual structure, and thematic evolution using KeyWords Plus™.

## 3. Results

### 3.1. Publication Year

**Figure 2:**
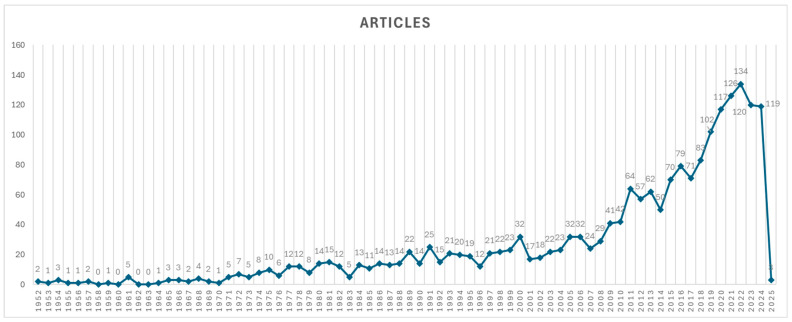
Annual distribution of articles according to year of publication. Note: The number of articles for 2025 includes only those published up to 26 February 2025, which explains the apparent decrease compared to previous years.

Figure 2 shows the evolution of the scientific production of water contamination by microorganisms, highlighting a continuous growth over the past decades, with greater intensity in the last five years. Of the 2000 articles evaluated, the majority were published in 2022, with 134 articles (6.70%), followed by the year 2021, with 126 articles (6.30%), and in 2023, with 120 articles (6.00%). The research was conducted considering the period from 1952 to 2025; however, the first relevant article identified was published in 1952, thus delimiting the period considered for analysis between 1952 and 2025.

### 3.2. Scientific Journals

**Figure 3:**
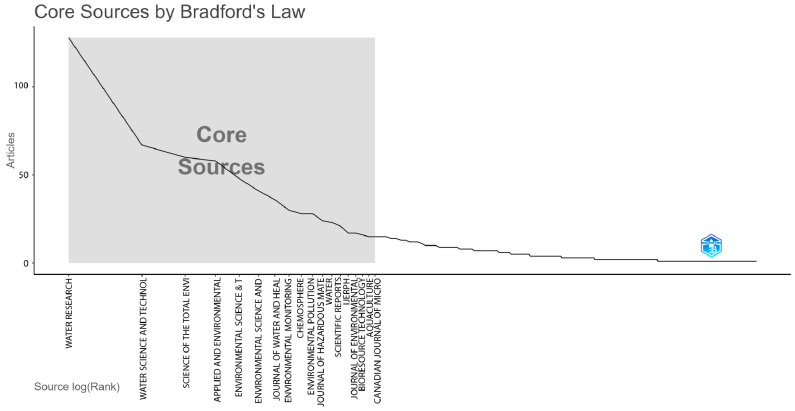
Dispersion of scientific knowledge according to Bradford’s law.

The analysis of publication sources on the topic was performed based on the application of Bradford’s Law (Figure 3), which identifies the most relevant journals in the field studied. Following the application of this law, it was found that the journal Water Research stands out as the main core source, with a total of 128 published articles.

Other journals that make up the central core of relevance include Water Science and Technology with 60 articles, Science of the Total Environment with 58 articles, Applied and Environmental Microbiology with 48 articles, and Environmental Science & Technology with 41 articles.

**Figure 4:**
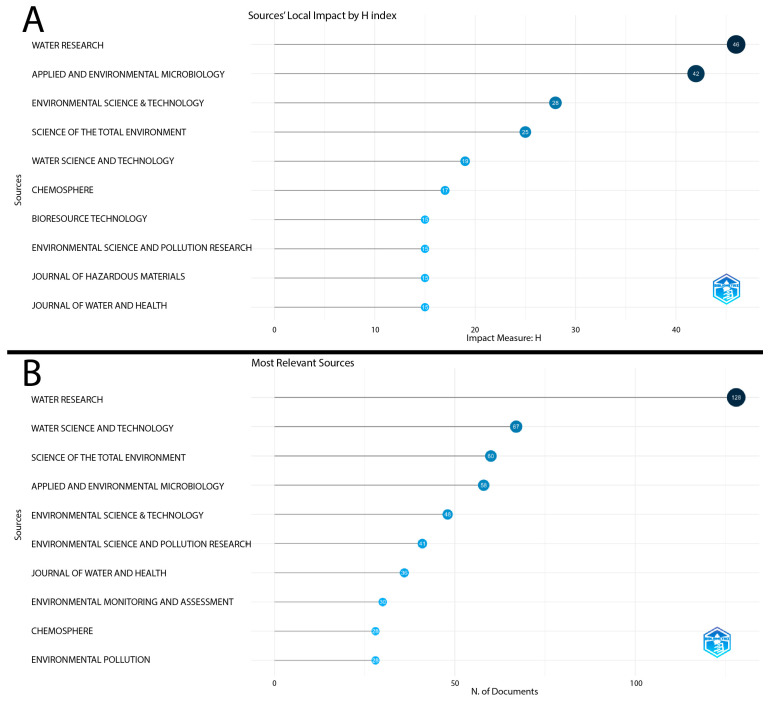
(**A**) Most cited scientific journals by local H-index. (**B**) Scientific journals with the highest number of publications on water contamination by microorganisms.

Another relevant aspect in the analysis of publication sources is the local impact measured using the H-index, which quantifies both the productivity and the impact of the articles published in each journal. The journal Water Research once again leads the ranking with an H-index of 46, followed by Applied and Environmental Microbiology with an H-index of 42 and Environmental Science & Technology with an H-index of 28, as described in Figure 4.

### 3.3. Authors’ Production

**Figure 5:**
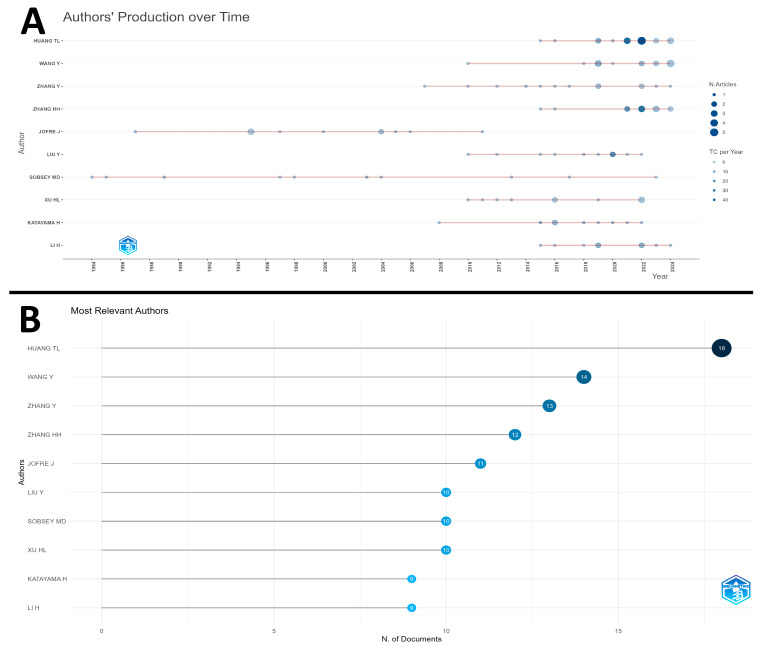
(**A**) Authors’ scientific production over time. (**B**) Top authors with the highest number of publications on water contamination by microorganisms.

The analysis of author productivity over time, as demonstrated in Figure 5A, shows that the size of the bubble is proportional to the number of published articles, while the color intensity reflects the total number of citations (TC) per year. Authors with larger and darker bubbles indicate higher productivity and academic impact, respectively. The authors Huang TL, Wang Y, Zhang Y, Zhang HH, and Jofre J stand out with a high number of publications, while Jofre J and Sobsey MD exhibit greater impact in terms of citations, even with a relatively smaller number of articles.

Figure 5B shows the graph of the most relevant authors, listing the leading researchers in terms of the number of publications. Author Huang TL leads with 18 articles, followed by Wang Y (14), Zhang Y (13), Zhang HH (12), and Jofre J (11).

### 3.4. Affiliate Institutions

**Figure 6:**
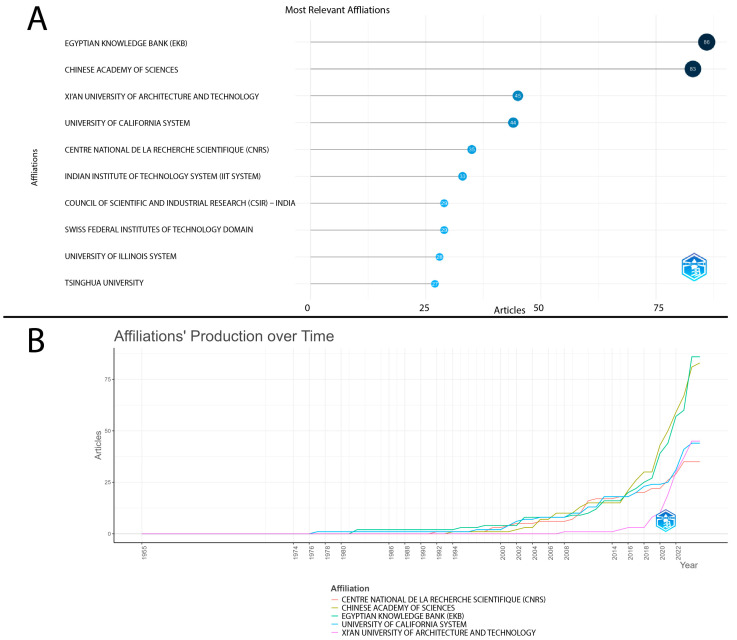
(**A**) Institutions with the highest number of publications (**B**) Temporal evolution of scientific production by the most relevant affiliations.

Figure 6 shows the temporal evolution of scientific production from the most relevant institutions in the field. It can be observed that the five institutions with the highest cumulative production over the years are as follows: the Egyptian Knowledge Bank (EKB) with the highest number of published articles (86), followed by the Chinese Academy of Sciences (83), Xi’an University of Architecture and Technology (45), the University of California System (44), and Centre National de la Recherche Scientifique (CNRS) (35).

Since 2015, there has been a significant increase in article production, especially by EKB and the Chinese Academy of Sciences, which stand out as the institutions with the highest number of recent publications (Figure 6B).

### 3.5. Countries and Collaborations

**Figure 7:**
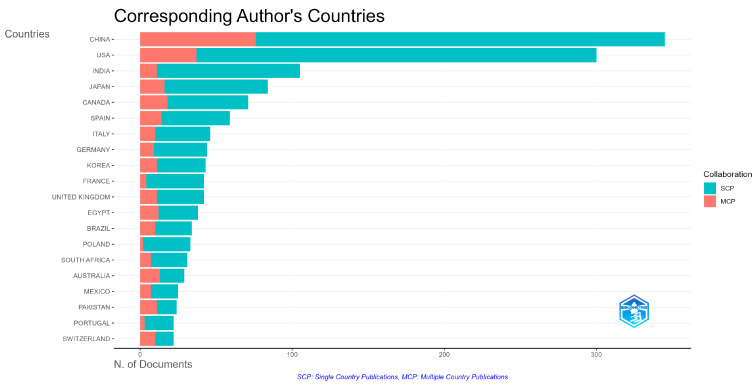
Distribution of corresponding authors’ affiliation countries and international collaborations (SCPs and MCPs).

Figure 7 shows the countries of affiliation of the corresponding authors, highlighting both publications made by a single country (SCPs—single-country publications) and international collaborations (MCPs—multiple-country publications).

The results indicate that China leads in the number of both isolated and collaborative publications. The United States also shows high scientific production, with a significant presence in international collaborations, while maintaining substantial unilateral production. Countries such as India and Japan demonstrate relevant scientific production, with a predominance of internal publications, while Canada, Spain, Italy, and Germany stand out particularly for their international collaborations.

### 3.6. Most Cited Articles

A total of 2000 articles were cited 50,913 times, with an average of 25.47 citations per item. The 12 most cited articles ranged from 475 to 249 citations (Table 1). These articles were published in nine different journals between 1991 and 2018.

### 3.7. Conceptual Structure

**Figure 8:**
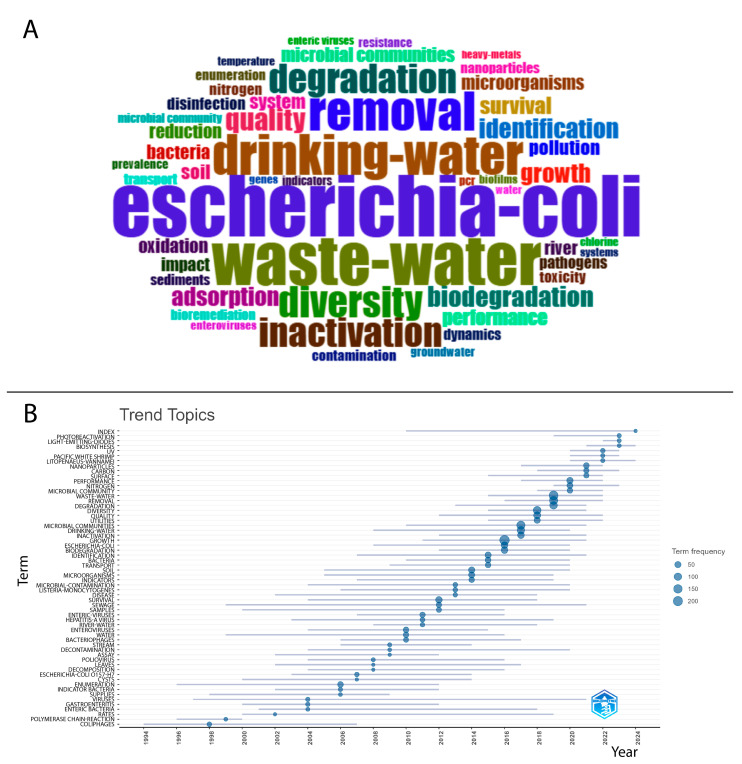
(**A**) Co-occurrence network of the most frequent keywords. (**B**) Evolution of trend topics in publications over time, based on keyword frequency.

In bibliometric research, keywords can summarize the focus of articles and determine which subjects are being addressed, that is, their conceptual structures [30]. Thus, to demonstrate the conceptual structure of the articles, the 50 most frequent KeyWords Plus™ were used to create Figure 8.

In part A, the figure displays a word cloud composed of the 50 most frequent KeyWords Plus™ found in the analyzed publications. The prominence of terms such as “Escherichia coli”, “waste-water”, “drinking-water”, “removal”, “degradation”, and “inactivation” reflects the central themes addressed in the scientific literature on water microbiological contamination. 

The size of each term represents its frequency, enabling a visual understanding of which topics have been most explored by researchers. Notably, terms related to microbiological indicators (e.g., Escherichia coli), water matrices (e.g., wastewater, drinking water), and treatment strategies (e.g., removal, inactivation, biodegradation) suggest a strong emphasis on monitoring and remediation approaches.

In part B, the temporal evolution of emerging research topics is presented, showing a sharp increase since the 2000s, with notable terms such as wastewater, drinking water, *Escherichia coli*, and degradation.

**Figure 9:**
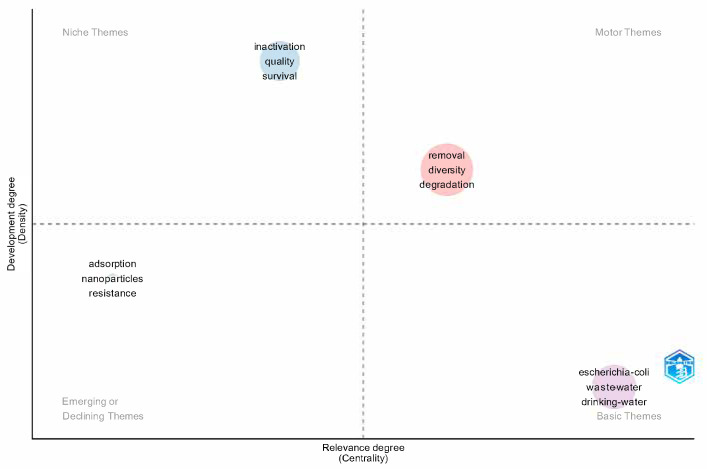
Thematic map showing clusters of keywords based on centrality (relevance) and density (development). The quadrants represent the following: motor themes (**top right**), niche themes (**top left**), emerging or declining themes (**bottom left**), and basic themes (**bottom right**). Examples include the following: removal, diversity, and degradation (motor); inactivation, quality, and survival (niche); adsorption, nanoparticles, and resistance (emerging); and Escherichia coli, wastewater, and drinking water (basic).

The thematic map (Figure 9), which classifies topics based on their centrality and density, identifies four quadrants: basic themes, motor themes, emerging or declining themes, and niche themes [31]. Basic themes include *Escherichia coli*, wastewater, and drinking water. Motor themes, which have high centrality and density, include removal, diversity, and degradation. Emerging or declining themes include adsorption, nanoparticles, and resistance. Finally, niche themes, such as inactivation, quality, and survival, represent specific areas with high density but low centrality.

## 4. Discussion

The bibliometric analysis that was conducted revealed a robust overview of the scientific production related to water contamination by microorganisms, highlighting a significant increase in the volume of publications over the past two decades. This growth reflects the intensification of research efforts aimed at water quality and public health risks, driven by emerging and critical topics such as climate change, antimicrobial resistance, and environmental contaminants [1,32].

In recent years, there has been a growing interest in investigating the impacts of climate change on human health, including outbreaks caused by drug-resistant pathogens and emerging contaminants. Extreme weather events, such as droughts followed by heavy rainfall, have been associated with water contamination outbreaks, as exemplified by the *Escherichia coli* O157:H7 outbreak in the United Kingdom in 2022, related to adverse weather conditions that contributed to the contamination of agricultural crops [33,34].

The search for solutions to mitigate the effects of climate change and the increase in antimicrobial resistance aligns with the United Nations Sustainable Development Goals (SDGs), which promote health and well-being (SDG 3), clean water and sanitation (SDG 6), and action against global climate change (SDG 13) [4,35]. The identification of new emerging contaminants capable of destroying sensitive microorganisms and favoring more resistant ones underscores the importance of robust research on risk assessment and the search for effective interventions [29].

The evolution of this field can also be observed, showing a growing scientific interest and increased investments in research motivated by critical events that challenge public health and global policy agendas.

The analysis of the most relevant journals revealed a strong concentration of scientific production in specific core sources, particularly highlighting the journals Water Research (Journal Citation Reports™ 2023: 11.5), Water Science and Technology (Journal Citation Reports™ 2023: 2.5), and Science of the Total Environment (Journal Citation Reports™ 2023: 8.2). These journals stand out both in terms of local impact, measured by the H-index, and in the volume of publications related to the topic of water contamination by microorganisms.

This strategic choice reflects researchers’ efforts to maximize the impact and visibility of their scientific findings, utilizing established and specialized outlets to disseminate their results widely and effectively.

The application of Bradford’s Law reinforces the concept of core sources, highlighting that a few journals concentrate most of the published articles on water contamination. This concentration suggests that researchers prioritize high-impact journals to ensure the visibility and credibility of their investigations, taking into account not only academic rigor but also the breadth of international impact.

When analyzing author productivity over time, it is observed that Huang TL stands out as the most productive, with a total of 18 publications. Next are Wang Y, Zhang Y, Zhang HH, and Jofre J. However, although Huang TL leads in the volume of publications, his scientific impact, measured by the number of citations, does not proportionally accompany the number of published works. On the other hand, authors like Jofre J and Sobsey MD, despite having fewer articles, are widely cited, demonstrating the significant impact of their research on the academic community.

The trajectory of scientific production also reveals distinct profiles among authors. While Huang TL and Wang Y maintain continuous publication over the years, reflecting consistent engagement with the topic, Jofre J and Sobsey MD exhibit specific productivity peaks, possibly related to collaborative projects or punctual initiatives with significant repercussions.

Notably, Sobsey MD emerges as a pioneer in the field, with publications dating back to the 1980s, establishing the theoretical and methodological foundations for the subsequent development of the field. His influence is evident in subsequent studies conducted by authors who gained prominence in later periods. Consequently, the combination of high productivity and consolidated impact through citations reflects the academic relevance of these researchers.

The analysis of the most relevant institutions in scientific production on water contamination by microorganisms highlights a strong concentration of publications in centers of excellence, especially after 2015, when a significant increase in academic output is observed.

The institutions that stand out the most in terms of cumulative production volume are the Egyptian Knowledge Bank (EKB), with 86 published articles, followed by the Chinese Academy of Sciences, Xi’an University of Architecture and Technology, University of California System (44), and Centre National de la Recherche Scientifique (CNRS). This scenario highlights the predominance of Asian and North American institutions in advancing research on water contamination and water quality.

The EKB stands out as a governmental initiative of Egypt aimed at consolidating a national knowledge platform, promoting open access and the dissemination of scientific research. This effort reflects the country’s commitment to academic advancement and the democratization of knowledge.

On the other hand, Chinese institutions, such as the Chinese Academy of Sciences and Xi’an University of Architecture and Technology, represent China’s robust investment in science and technology, consolidating the country as a global leader in academic production. This leadership results from governmental policies that encourage research and promote international partnerships, expanding the visibility and global impact of the studies that were conducted.

China leads both in single-country publications (SCPs) and international collaborations (MCPs), highlighting the rapid growth of its universities and the strengthening of scientific networks on the global stage.

North American institutions, such as the University of California System, also stand out for their relevance in scientific production focused on water quality monitoring and the development of treatment strategies. Additionally, the CNRS, from France, consolidates itself as a reference for its interdisciplinary approach and international collaborations, enhancing the impact of its publications and strengthening knowledge exchange at a global level.

An analysis of the 12 most cited articles on water contamination by microorganisms revealed three predominant thematic axes: disinfection and treatment technologies [18,19,23,24,27], microbiological water quality indicators [21,25,26,28], and antimicrobial resistance and emerging contaminants [20,22,29]. Although these studies represent only a fraction of the analyzed database, they were selected for their high impact and fundamental contributions to the advancement of the field. They not only synthesize the main challenges in the area but also illustrate methodological and technological milestones that have shaped the global research agenda.

In the disinfection and treatment axis, there is a noticeable shift from conventional methods, such as chlorination, to more sustainable and innovative approaches, such as solar-assisted photocatalysis and the use of nanomaterials. The work by Frederik Hammes et al. (2008) [18], the most cited in the sample, introduced the use of flow cytometry for microbiological monitoring, representing a turning point in the rapid and continuous detection of bacteria in water supply systems. This innovative technique has enabled a significant advancement in the rapid and accurate detection of bacteria, allowing for a quick response in water quality control. The study highlights the importance of technologies that provide continuous and real-time monitoring, contributing to the effective management of microbiological risks in supply systems.

These findings are consistent with the topics classified as “motor themes” in the thematic map, such as removal, diversity, and degradation, reinforcing their centrality and conceptual density in the literature.

In the second axis, focusing on microbiological indicators, the studies highlight the persistence of Escherichia coli as the main marker of fecal contamination and the use of molecular tools for pathogen detection. These aspects correlate with the “basic themes” identified in the bibliometric research, such as drinking water, wastewater, and *E. coli*, indicating that these topics remain essential to the field and are widely addressed in an interdisciplinary manner.

In the axis concerning antimicrobial resistance and emerging contaminants, the articles highlight the emergence of new public health threats, often associated with the indiscriminate use of antibiotics and the presence of pharmaceutical residues in water bodies. The recognition of microbial resistance as a global challenge aligns with the “emerging themes” identified in the thematic map, such as resistance and nanoparticles. This trend points to the need for integration among public health policies, environmental management, and technological innovation.

Another relevant study is that of Chang et al. (1994) [19], which investigated the use of photocatalysis with TiO_2_ for solar-assisted water disinfection, presenting promising results for regions with high solar radiation incidence and a lack of traditional technological resources. The bactericidal efficacy of these approaches reinforces their practical applicability and their relevance in the current context of seeking sustainable and effective solutions.

Furthermore, other highlighted studies include the application of modern technologies for the remediation and monitoring of water quality, reflecting the continuous effort of the scientific community to address contemporary challenges related to water quality management [6,25,29,36,37].

In this context, the journal Environmental Science & Technology (Journal Citation Reports™ 2023: 10.9) stands out as the most recurrent publication among the most cited articles in this analysis. This can be attributed to the methodological rigor required by the journal and its multidisciplinary approach, integrating environmental sciences, analytical chemistry, and sustainable water treatment technologies.

Furthermore, it is important to highlight that the articles published in this journal frequently address emerging topics, such as the use of new technologies for pathogen control and the evaluation of emerging contaminants. This approach justifies the prominence of the journal among the most cited articles in the field of water contamination, emphasizing the importance of investing in innovative and robust methodologies that ensure water safety in the face of contemporary global demands [38,39].

The analysis of the keyword co-occurrence network revealed two main thematic clusters related to the topic. The first cluster, identified by the color blue, encompasses terms associated with degradation processes, diversity, and removal, highlighting microbial communities, heavy metals, and sediments. This configuration reflects the scientific interest in addressing pollutant degradation and microbial biodiversity preservation as strategies to improve water quality.

On the other hand, the second cluster, represented by the color red, is centered on *Escherichia coli* and wastewater, emphasizing topics such as inactivation, disinfection, and contamination. The prominent presence of *E. coli* reflects the concern with its control, as this pathogen is one of the main indicators of fecal contamination in supply systems [40].

The temporal evolution of emerging topics demonstrated a significant increase in research from the 2000s onwards, driven by the intensification of global concerns regarding water safety and pathogen control. Terms such as wastewater, drinking water, and degradation stand out, reflecting the growing demand for technological and practical solutions that ensure water potability in a context of accelerated urbanization and climate change. The search for new monitoring and treatment methods aligns with international efforts, particularly the Sustainable Development Goals (SDGs), which establish targets for the sustainable management of water resources [41].

The thematic map provides a comprehensive view of the main topics addressed, organizing them into four quadrants that indicate different degrees of centrality and density. Basic themes, such as *Escherichia coli*, wastewater, and drinking water, represent consolidated and essential subjects for the field of study, reflecting their persistence on the research agenda. In contrast, motor themes, such as removal, diversity, and degradation, exhibit high centrality and density, standing out as areas of great relevance and scientific impact, especially in studies aimed at improving treatment and remediation methods.

In contrast, emerging or declining themes, such as adsorption, nanoparticles, and resistance, indicate innovative approaches that still lack widespread acceptance or consolidated application, suggesting that technological advancements may consolidate or modify the approach to these topics in the future. On the other hand, niche themes, such as inactivation, quality, and survival, have high density but low centrality, indicating that, although they are relevant in specific contexts, they do not exert significant influence on the current body of research.

The evolution of the field of water contamination reflects a dynamic and complex movement, driven by technological advancements and the growing demand for efficient and sustainable solutions [42]. In recent years, microbiological control strategies have begun to incorporate more sophisticated techniques, such as the use of biotechnologies and the application of advanced materials, including nanoparticles, to enhance disinfection processes and water quality monitoring [4].

This evolution has been consolidated through the integration of multidisciplinary approaches that promote public health protection and meet global demands for water safety, especially in vulnerable regions with inadequate infrastructure [40]. The application of emerging technologies, such as the use of nanoparticles and real-time monitoring, has proven promising in mitigating microbiological risks and ensuring water potability [43].

Such strategies offer greater precision in pathogen detection and faster responses to epidemiological outbreaks, enabling more effective and evidence-based interventions. Additionally, the combination of traditional methods with new technological approaches has the potential to optimize treatment processes and ensure the maintenance of water quality [43].

Another contemporary challenge that deserves attention is microbial resistance, which has been intensifying due to inadequate water management practices and environmental degradation. This issue requires a continuous effort from the scientific community to develop methodologies that not only detect pathogenic microorganisms but also assess their adaptability and resistance in different environmental contexts [44].

To this end, it is essential to adopt strategies that integrate theoretical knowledge with integrated and sustainable water resource management practices, promoting a balance between technological innovation and environmental sustainability.

## 5. Limitations

This study involved some limitations that must be considered. First, the analysis was based exclusively on the Web of Science (WoS) Core Collection database. Although this is a widely recognized and high-quality platform, it does not include the full range of scientific literature, particularly from regional journals or gray literature. Second, the search strategy was restricted to article titles to improve specificity, which may have excluded relevant studies indexed under broader metadata fields. Third, data collection was conducted up to 26 February 2025; therefore, the number of publications in 2025 is partial and should not be interpreted as indicative of a declining trend. These factors may have influenced the completeness and generalizability of the findings.

## 6. Conclusions

This bibliometric analysis showed that most of the 2000 evaluated articles were published in 2022, with a concentration in high-impact journals such as *Water Research*, *Water Science and Technology*, and *Science of the Total Environment*. The most productive institutions were the Egyptian Knowledge Bank and the Chinese Academy of Sciences, highlighting China’s strong performance in both national and international scientific collaboration.

The main research topics identified include innovative methods for water decontamination, microbiological quality assessment, and strategies to mitigate antimicrobial resistance. Thematic mapping revealed a focus on the identification and inactivation of waterborne pathogens, supported by ecological and biotechnological approaches.

One relevant finding is the emerging integration of real-time monitoring technologies with artificial intelligence, which offers potential to predict contamination events and improve responses, especially in vulnerable settings.

Additionally, the study reinforces the relevance of this topic for health education and professional training by supporting the inclusion of water microbiology in academic curricula as a public health and environmental safety priority.

## Figures and Tables

**Table 1:** Ranking of the most cited published articles on water contamination by microorganisms.

Rank	Authors (Year), Journal	Title	Total Citations
1	Frederik Hammes et al. (2008) [18], Water Res	Flow-cytometric total bacterial cell counts as a descriptive microbiological parameter for drinking water treatment processes	475
2	Chang. Wei et al. (1994) [19], Environ Sci Technol	Bactericidal Activity of TiO_2_ Photocatalyst in Aqueous Media: Toward a Solar-Assisted Water Disinfection System	436
3	Tanja Barac et al. (2004) [20], Nat Biotechnol	*Engineered endophytic* bacteria improve phytoremediation of water-soluble, volatile, organic pollutants	420
4	Peter Kämpfer et al. (1991) [21], Microb Ecol	Microbiological characterization of a fuel-oil contaminated site including numerical identification of heterotrophic water and soil bacteria	408
5	Chad W. McKinney et al. (2012) [22], Environ Sci Technol	Ultraviolet Disinfection of Antibiotic Resistant Bacteria and Their Antibiotic Resistance Genes in Water and Wastewater	396
6	Qi Bao et al. (2011) [23], J Colloid Interf Sci	Synthesis and characterization of silver nanoparticle and graphene oxide nanosheet composites as a bactericidal agent for water disinfection	381
7	Theresa A. Dankovich et al. (2011) [24], Environ Sci Technol	Bactericidal Paper Impregnated with Silver Nanoparticles for Point-of-Use Water Treatment	377
8	Ameet J. Pinto (2012) [25], Environ Sci Technol	Bacterial Community Structure in the Drinking Water Microbiome Is Governed by Filtration Processes	337
9	Michael A. Mallin et al. (2000) [26], Ecol Appl	Effect of human development on bacteriological water quality in coastal watersheds	301
10	Darryl H. Dvorak et al. (1992) [27], Biotechnol Bioeng	Treatment of metal-contaminated water using bacterial sulfate reduction: Results from pilot-scale reactors	286
11	P Payment et al. (1993) [28], Appl Environ Microb	*Clostridium perfringens* and somatic coliphages as indicators of the efficiency of drinking water treatment for viruses and protozoan cysts	255
12	SU HC, et al. (2018) [29] Sci Total Environ	Persistence of antibiotic resistance genes and bacterial community changes in drinking water treatment system: From drinking water source to tap water	249

## Data Availability

The data used in this bibliometric study were retrieved from the Web of Science™ Core Collection, accessed on 26 February 2025. No new datasets were generated.
